# Minimal Residual Disease Detection in Acute Lymphoblastic Leukemia

**DOI:** 10.3390/ijms21031054

**Published:** 2020-02-05

**Authors:** Aaron Kruse, Nour Abdel-Azim, Hye Na Kim, Yongsheng Ruan, Valerie Phan, Heather Ogana, William Wang, Rachel Lee, Eun Ji Gang, Sajad Khazal, Yong-Mi Kim

**Affiliations:** 1 Children’s Hospital Los Angeles, University of Southern California, 4650 Sunset Boulevard, MS #57, Los Angeles, CA 90027, USA; a.kruse@usc.edu (A.K.); nabdelaz@usc.edu (N.A.-A.); hyekim@chla.usc.edu (H.N.K.); yruan@chla.usc.edu (Y.R.); phanvale@usc.edu (V.P.); hogana@chla.usc.edu (H.O.); wsw253@nyu.edu (W.W.); lee646@usc.edu (R.L.); ejiang@chla.usc.edu (E.J.G.); 2 Department of Pediatrics Patient Care, Division of Pediatrics, The University of Texas MD Anderson Cancer Center, Houston, TX 77030, USA; SJKhazal@mdanderson.org

**Keywords:** minimal residual disease, acute lymphoblastic leukemia, B-cell acute lymphoblastic leukemia, T-cell acute lymphoblastic leukemia, flow cytometry, polymerase chain reaction, next-generation sequencing

## Abstract

Minimal residual disease (MRD) refers to a chemotherapy/radiotherapy-surviving leukemia cell population that gives rise to relapse of the disease. The detection of MRD is critical for predicting the outcome and for selecting the intensity of further treatment strategies. The development of various new diagnostic platforms, including next-generation sequencing (NGS), has introduced significant advances in the sensitivity of MRD diagnostics. Here, we review current methods to diagnose MRD through phenotypic marker patterns or differential gene patterns through analysis by flow cytometry (FCM), polymerase chain reaction (PCR), real-time quantitative polymerase chain reaction (RQ-PCR), reverse transcription polymerase chain reaction (RT-PCR) or NGS. Future advances in clinical procedures will be molded by practical feasibility and patient needs regarding greater diagnostic sensitivity.

## 1. Introduction

### 1.1. Description of Minimal Residual Disease

Minimal residual disease (MRD) in acute lymphoblastic leukemia (ALL) is the presence of post-therapeutic (chemotherapy, immunotherapy, or radiotherapy) leukemia cells within the bone marrow and more rarely in peripheral blood circulation. MRD cells can be profiled as remnants of pretreatment originator ALL cells or as transformed secondary ALL. Transformed secondary ALL cells are distinguishable from pretreatment originator ALL cells by their unique rearrangement patterns and identifiable immunoglobulin (Ig) and T-cell receptor (TCR) gene variations. Secondary ALL cannot be traced back to an identical pretreatment originator ALL cell and might represent 5–10% of cases [1,2]. Relapsed ALL cells can also be traced back to other early B or T cell transformations before evolving into overt leukemia.

The primary clinical purpose for monitoring MRD is to determine the response to treatment and the risk of leukemia relapse. MRD levels are also used to modify the intensity and duration of chemotherapy (which may include allogeneic stem cell transplantation) and to create risk profiles for patients based on measured clearance of leukemic cells and post-treatment probability of disease relapse correlated to MRD levels. Relapse prognostics are determined by measuring MRD levels in patient samples at various time points during and after a chemotherapy regimen. MRD levels are evaluated from patient bone marrow aspirates, which are obtained at multiple independent time points throughout the treatment regimen. Cellular MRD counts have general prognostic value at the cutoff level of 0.01% MRD cells (10^−4^): meaning 1 MRD cell in 10,000 cells out of all bone marrow mononuclear cells within a specimen. The prognostic limit of 0.01% is based on the immunohistochemical detection limits of 3–4-color flow cytometers. The clinical significance of the 0.01% MRD cutoff level is that when a patient has cellular MRD levels ≥0.01% in a bone marrow sample at important measurement time points during therapy, the patient will have a significantly higher risk for leukemia relapse than if MRD levels are less than 0.01% [3,4,5]. Data also suggest that the higher the MRD value (e.g., MRD > 1%) at the end of the induction phase of chemotherapy, the higher the risk of relapse and the lower the survival rate [6].

MRD levels are also a primary prognostic determinant of post-therapeutic progress, and measurements are used by clinicians as a tool for risk assignment strategies and therapy decisions. MRD measurements at specific clinical endpoints show statistical significance as a consistent variable in treatment strategy decisions in data from major clinical cohorts in Europe and the United States (US).

Standardized procedures for relapse treatment call for early adjustments in therapy intensity and medications because of potentially severe side effects throughout the 2–3-year period of treatment. Therapy adjustment decisions made during the induction period during the first 30 days of treatment include intensification of treatment for patients showing low treatment response as measured by detectable MRD levels, whereas low-risk patients with no MRD (≤0.01%) at the end of induction therapy should be evaluated for therapy reduction to prevent chemotherapy sequelae, which can include secondary malignancies, cardiomyopathy, endocrinopathies, and neuropsychological problems among other long term effects. MRD guidance in therapy decisions demonstrates improved patient outcomes in both therapy reduction and therapy intensification [7,8,9] and serves as a prognostic indicator and therapy modification variable in stem cell transplantation [10,11,12,13,14,15]. In the US, the Children Oncology Group (COG) protocol AALL0932 “Treatment of Patients with Newly Diagnosed Standard Risk B-Lymphoblastic Leukemia (B-ALL) or Localized B-lineage Lymphoblastic Lymphoma (B-LLy)” used day 8 induction peripheral blood MRD in risk stratifying patients with the aim of de-intensification of therapy in low-risk leukemia children.

### 1.2. Genetic Descriptions of B-Cell Acute Lymphoblastic Leukemia and Minimal Residual Disease

Upon transformation from a hematopoietic progenitor cell, the leukemic stem cell (LSC) will follow a committed lineage pathway and differentiate into a mutated (neo)-colony forming unit-lymphocyte and replicate into developmentally arrested pre-B-cell lymphoblasts and also pre-T-cell lymphoblasts [16]. The replication of a progenitor cell into a B-cell ALL (B-ALL) may arise either from a mutated multipotent progenitor or a committed progenitor cell already in the defined lymphocyte lineage stage. B-ALL (and T-cell ALL) have clonal rearrangements in the Ig and T-cell receptor genes and express surface glycoproteins and antigen receptors similar to pre-B-cell and pre-T-cell lymphocytes. The most important mutation outcomes from LSCs are the capacity for unlimited self-renewal and developmental arrest at the pre-lymphocyte developmental stage. These cell profiles can be the result of several factors, including the aberrant expression of proto-oncogenes, chromosomal translocations that express fusion genes that encode transcription factors and active kinases, and post-mitotic aneuploid cell development [17]. The basic immunophenotype of B-ALL displays positive staining in 95% of cells for terminal deoxytidyl-transferase (TdT) type DNA polymerase, and B-cell markers CD19 and CD10 (except very immature B-ALL). Mature pre-B cell lymphoblasts express CD10, CD19, CD20, and IgM heavy chain (μ chain) in the cytoplasm or as an early surface marker [18]. Clonal replication results in leukemia if more than 25% of the nucleated cells in the marrow compartment are B-cell lymphoblasts or if there are less than 20% lymphoblasts but the patient presents with any of the following known recurring cytogenic abnormalities: hypodiploidy; hyperdiploidy; translocation t(12;21)(p13;q22) *ETV6-RUNX1* (formerly *TEL-AML1*); t(9;22)(q34;q11.2) *BCR-ABL1*; t(5;14)(q31;q32) *IL3-IGH*; t(1;19)(q23;p13.3) *TCF3-PBX1*; and *MLL* rearrangements including t(4;11), t(11;19), t(9;11) [19]. These translocations cause fusion genes encoding chimeric transcription factors that alter signaling cascades and modify the normal expression of many genes. In addition to fusion genes, other cooperative mutations within the chromosomal structure are required to fully alter the progenitor cells to create the leukemia condition. The prognosis for different subtypes of B-ALL varies and shows consistent outcomes as determined by historical analysis (Table 1).

The general strategy for treating ALL involves the use of chemotherapy to eradicate leukemia cells in the bone marrow and peripheral circulation. Treatment regimens for childhood and adult ALL rely on similar protocols, which consist of three consecutive phases and include in order: (1) remission-induction therapy; (2) intensification/consolidation therapy; and (3) continuation treatment.

Treatment strategies for B-ALL have resulted in up to a 90% cure rate in children but show only 30–40% remission results in adult patients [20]. Chemotherapy sequelae can include secondary leukemias, tumors, cardiomyopathy, and neuropsychological problems, among other symptoms.

## 2. Prognostic Value of MRD

The prognostic value and clinical significance of MRD quantification relating to ALL were first investigated in the 1990s in multiclinic centers in Europe and the United States. Research groups concluded that MRD assessment should be made early during treatment (typically the end of induction phase) and at multiple time points after using flow cytometry (FCM) and/or polymerase chain reaction (PCR) analyses of bone marrow aspirates samples. Related studies during this period showed that MRD status was a reliable and independent indicator of the risk of future relapse [21,22,23,24]. Cave et al. [21] observed that PCR was successfully used to identify leukemic cells in the bone marrow after induction chemotherapy, and residual leukemia at a level of 10^−3^ or higher was found to be highly predictive of relapse, and leukemia cell levels above 10^−2^ showed an even higher increase in relapse rates in patients. Coustan-Smith et al. [22] used flow cytometry to examine leukemia clearance in childhood relapse cases. Van Dongen et al. [23] used PCR to study MRD levels in patients during relapse therapy and found that MRD levels ≥10^−2^ were highly associated with relapse. Relapse prognostics are most significant when MRD cell levels exceed 0.01% at the end of induction therapy [25,26]. The measurement of MRD levels at different time points during therapy is now used routinely as a tool to risk-stratify patients, make treatment decisions, and gauge therapy effectiveness [25,27,28,29]. The evaluation for MRD is not used only to evaluate the response to treatment and risk of relapse during standard therapy only; it has an invaluable prognostic value after other therapeutic modalities for acute leukemia, including allogeneic hematopoietic stem cell transplantation [30]. Recently, new emerging data on the value of MRD using next-generation sequencing post chimeric antigen receptor T cell therapy can help predict the risk of disease relapse, which has therapeutic implications on which patient population may benefit from remission consolidation with allogeneic hematopoietic stem cell transplantation [31].

## 3. Phenotypic and Genetic Detection of MRD

**Figure 1:**
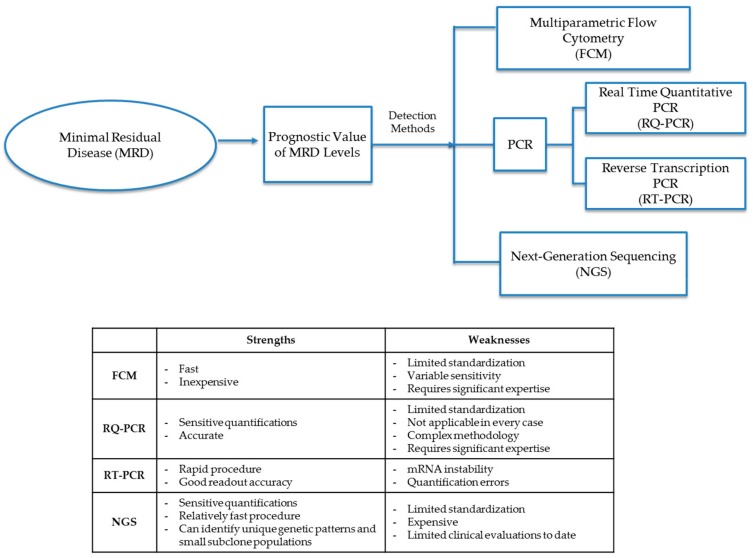
Detection methods for minimal residual disease (MRD). Methods to diagnose MRD either through phenotypic marker patterns or differential gene patterns through analysis by FCM (flow cytometry), PCR (polymerase chain reaction), RQ-PCR (real-time quantitative polymerase chain reaction), RT-PCR (reverse transcription polymerase chain reaction) or NGS (next-generation sequencing).

The MRD cellular level in diagnostic leukemia relapse samples is the primary variable and prognostic indicator of future treatment decisions and outcomes. Chemotherapy agents (including steroids) not only help to eliminate leukemic cells but can also give rise to epigenetic mutations in remaining leukemia cells. Treatment agents may leave small populations of leukemic MRD cells, which may either be clones of pretreatment leukemia progenitor cells or populations of mutated leukemia cells that either have different cellular markers than that of original diagnostic leukemia cells or have mutated genotypes that display differential expression of Ig and TCR gene patterns. Molecular detection methods for MRD identify cells either through patterns of phenotypic markers or differential gene expression through analysis by FCM, PCR, or next-generation sequencing (NGS) (Figure 1 and Table 2).

### 3.1. Multiparametric Flow Cytometry

MRD detection by phenotype identifies surface antigen markers and can differentiate normal bone marrow lymphocytes and myeloid cells from mutated leukemic progenitor cells. Multiparametric flow cytometry (FCM) detection of leukemic MRD cells begins with the assessment of pretreatment diagnostic panels (original immunophenotype) of patient leukemic cells. Pretreatment leukemia cells display different cellular marker combinations from other bone marrow cells and serve as the cellular subpopulation identifier for a patient’s diagnosis based on the known leukemia subtypes. Later, if a patient has a relapse, bone marrow aspirations collected at designated intervals during chemotherapy are analyzed by FCM for MRD and compared to pretreatment cell panels.

To identify MRD cells, immunofluorescent tagged antibodies and ligands specific to leukemia cell surface markers are mixed with the aspirate cell sample and run through a FCM protocol to generate a dot plot cellular sample profile. B-cell maturation from a committed precursor cell is a multistep process that can be monitored at distinct time points to verify the acquisition and loss of cell surface markers. Through coordinated FCM readings, MRD cells can be identified through comparison with pretreatment cell panels or cataloged patterns [38,39,40]. Leukemia immunophenotypes have been extensively researched with MRD cellular patterns described by synchronous antigen expression, cross-lineage antigen expression, antigen overexpression/underexpression, and light scatter aberrancies [41]. Normal pre-B cells express designated cell markers that are differentially expressed in leukemic pre-B-cell phenotypes, and MRD derivations often reveal differential up- and downregulation of leukemic phenotypes in a time-dependent manner.

Early immunophenotype investigations of MRD involved two- and three-color flow cytometers, which offered patient-specific immunophenotyping in which cellular markers were measured after patient diagnosis and used to baseline later diagnostic MRD measurements. Advances in marker identification of pre-B cells have helped to create standardized profiles of cellular antigen receptor combinations at different stages of development with lymphoblast subsets identified by CD10, CD20, CD22, CD19, CD34, CD38, CD45, and CD58 combinations that allow for pattern recognition in FCM plots in the aberrant regions indicating MRD cellular phenotypes [39,42,43]. The adoption of four- to six-color flow cytometers further enhanced the labeling capacity of cells by using multiple fluorochrome markers, thereby increasing MRD profiling capabilities and improving MRD detection levels to 10^−4^ cells, thereby providing high concordance with PCR measurements [44]. Typical MRD measurements at day 15 of treatment will show high MRD/ALL levels often two logs higher than the statistically significant 0.01% relapse risk threshold, and aspirates from day 33 and 78 will normally demonstrate decreased MRD levels due to apoptosis and cell clearance.

Efforts in flow cytometer standardization in multicenter clinics rely on standard procedures for the use of monoclonal antibodies. Standard clinical procedures for MRD measurement using FCM have been made possible through European standardization protocols developed in multinational studies, such as AIEOP-BFM-ALL 2000 and BIOMED-1 [45,46].

Laboratories have increasingly transferred flow cytometry capabilities to eight-color flow cytometers [47] and even up to twelve-color readout capacities. Coordinated multiclinical efforts in European laboratories involving cross-platform instrument diagnostics and hematological malignancy classifications have led to standardized measures in flow cytometer machine calibration and lymphocyte immunophenotype markers in accordance with WHO standards [48,49]. Traditional FCM has relied on personalized patient sample analysis. Gating cross-platform standards have allowed for standardized immunophenotype identification and analysis. In Europe, as of 2017, a majority of flow cytometer manufacturers have cross-calibrated their multiple instruments to enable the measurement and data generation of the 8-color panels in a standardized way [50]. Modern eight-color flow cytometer machines are also capable of visualizing individual cells based on marker readouts [51].

An example of a FCM assay of relapsed MRD can be visualized by staining MRD-positive and MRD-negative patient samples for comparison and displaying differentiation of cell markers indicative of MRD-positive and MRD-negative samples. MRD can show phenotypic shifts between diagnostic and relapse samples of nearly 40–70% in precursor B-ALL [52]. MRD cells display differential presentation of cellular markers over time compared to normal B cells; however, treatment samples of MRD measured by FCM taken during induction phase relapse therapy can be reliable because, in most cases, MRD cell populations generally resemble diagnostic leukemic phenotypes. In the case of leukemic relapse phenotypes that are measured before therapy, which are different from original diagnostic leukemia, secondary ALL can still be detected from these progenitor cell types. MRD phenotypes will show markers similar to relapsed leukemia cells saved for reference from original diagnostic cells or can be identified through profile databases in the case of secondary ALL. In contrast to the four-color flow cytometers, which have the capacity to measure MRD up to the important diagnostic level of 0.01%, 8–12 color flow cytometers can normally measure MRD levels up to 0.001% cells or 1 MRD cell in 100,000 cells in a bone marrow specimen with even higher sensitivity [43,53].

### 3.2. Polymerase Chain Reaction

Polymerase chain reaction (PCR) provides quantitative MRD measurements extrapolated from amplification cycles of a given MRD DNA sample. The genetic targets of MRD cellular quantification include Ig and TCR gene rearrangements, breakpoint fusion regions of chromosome translocations, fusion gene transcripts, and other aberrant genes, including *FLT3-ITD*, *WT-1*, *HOX1 1L2*, and other transcripts. PCR detection of ALL MRD reaches a general sensitivity of 0.001%. PCR quantification for MRD requires 10^6^ cells obtained from a patient bone marrow sample.

Two methods of PCR are commonly used in MRD analysis. Real-time quantitative PCR (RQ PCR) allows for the quantification of DNA amplification products during the exponential phase of cycling by using fluorescent probes that emit fluorescence at critical points of cycling. A scalable signal emitted at the first readout level increases with every additional amplification cycle until a maximal readout is reached. Fluorescent probes that can be used in RQ-PCR include SYBR Green I, hydrolysis probes, and hybridization probes, all of which emit quantification fluorescence at breakthrough DNA concentration levels [23,40,54,55]. Reverse transcription PCR (RT-PCR) is a second common technique in MRD analysis in which fusion gene transcripts and other transcripts are processed through mRNA reverse transcription to yield cDNA exons that can be amplified through PCR cycling and yield quantifiable target sequence products through probe analysis.

Ig/TCR gene rearrangements are the most common quantification targets in ALL MRD analyses. Precursor B cells undergo variable (V), diversity (D), and joining (J) gene segment rearrangements in early development during mitosis, and the junctional regions in genes of heavy chain domains and TCR domains provide a “DNA fingerprint” in which a clonal progenitor cell will pass along to progeny cells. In the case of MRD, these fingerprints are identifiable reading frames experimentally cataloged and diagnostically comparable and differentiable from early diagnostic cell populations and relapse cell Ig/TCR genotypes. Although oligonucleotide primers are capable of synthesizing identifiable strands, cellular VDJ recombination tends to continue during therapy. VDJ junction regions are not oncogenically associated with the timelines of leukemia MRD mitotic proliferation, and consequently, samples of clonal MRD progenitor cells aspirated during therapy will display different junctional VDJ fingerprints over time. To circumvent this genotyping discrepancy which can lead to false-negative readings, PCR analysis monitors ALL MRD cell populations by using two or more independent Ig/TCR amplification targets during analysis [56,57,58,59]. In B-ALL and T-ALL, the junctional regions of Ig/TCR gene rearrangements are the fingerprint regions of malignant cells and are the tumor identifying target regions for MRD detection. B-ALL can be identified by fingerprint regions of the Ig heavy chain (IGH), Ig kappa light-chain (IGK), and Ig lambda (IGL) regions. T-ALL can be identified by TCR gamma rearrangements (TCRG), TCR delta rearrangements (TCRD), and TCR beta gene rearrangements (TCRB) [54,56].

DNA level fusion genes serve as the second primer target in MRD analysis and have distinct fingerprints at intron regions. Breakpoint fusion regions on a chromosome are unique to each patient without regard to the categorization of the specific ALL fusion gene, and as a result, individualized PCR analysis is performed for a given fusion gene subtype, particularly if intron identifiers are less than 10 kb in length for a given ALL subtype. Reading frames under 10 kb are valid identifiers for patients with MRD subtype designation, such as *BCR-ABL1*, but less common gene fusion breakpoints may have intron regions that span 200 kb in length, preventing analysis, and are, therefore, not diagnosable [54,57,60]. Fusion genes are good PCR targets because they are related to the oncogenic process and are durable elements of mitosis that are stable throughout the course of the disease. Fusion transcripts also serve as templates for MRD identification, and mRNA can be reverse transcribed to create a template strand for RT-PCR cycling [61]. mRNA can be identified with a limited set of primers and is related to oncogenesis since it is a translocation fusion product directly linked to a translocation genotype. The disadvantages of RT-PCR are that transcript amplification is not patient-specific, and therapy might affect expression levels, resulting in variable mRNA levels. Therapeutic drugs might also cause intracellular RNA instability [62,63,64].

MRD analysis in Ph+ ALL is difficult because of methodological differences related to the use of real-time quantitative PCR (qRT-PCR) in measuring BCR-ABL1 transcript levels. Recent guidelines for MRD in Ph + ALL patients were proposed by Pfeifer H et al. based on a study by the EURO-MRD consortium on standardization of qRT-PCR for the e1a2 BCR-ABL1 transcript in Ph + ALL, designed to overcome the lack of standardization of laboratory procedures and data interpretation. Standardized use of EAC primer/probe sets and centrally prepared plasmid standards had the greatest impact on reducing interlaboratory variability. Stringent application of technical criteria for assay quality and uniform criteria for data interpretation and reporting was essential [61]. The use of a cell-based secondary reference panel for BCR-ABL1 quantification for MRD analysis in chronic myeloid leukemia was recently published [58]. This may further improve the accuracy and consistency of MRD results.

During therapy, PCR is performed using patient bone marrow aspirations drawn at regular time intervals. Thirty amplification cycles require 1 μg of DNA, which provides 10^5^ to 10^6^ cells. MRD samples can also be obtained from peripheral blood; however, blood MRD levels are 10 times lower than levels in the bone marrow, requiring a PCR sensitivity of less than or equal to 10^−5^ [23,65,66,67]. Large libraries of primers are available for MRD identification, and patient-specific primers can be developed from diagnostic samples. In comparison to FCM, PCR is viewed as more laborious and expensive, although PCR has a sensitivity of one log higher than FCM. Since the 1990s, PCR has become a primary quantification assay for MRD analysis and is the primary protocol when immunohistochemical analysis fails to detect MRD.

Droplet digital polymerase chain reaction (ddPCR) is a relatively new technology used to identify MRD [68]. Like RQ-PCR, ddPCR uses Taq polymerase in DNA amplification techniques, and fluorescent probes are used to target DNA sequences in a sample; however, RQ-PCR provides only relative quantification, while dPCR provides absolute quantification of target DNA samples [69]. In ddPCR, reaction mixes are partitioned into approximately 20,000 droplets into separate reaction chambers and amplified [70]. Partitions are then subject to fluorescent probes, and each reaction chamber is examined for either the presence or absence of fluorescence. The frequency of positive amplifications is analyzed using the Poisson distribution to determine the template concentration [71]. The use of digital PCR (dPCR) was first published in 1999 in a paper by Vogelstein and Kinzler in which the feasibility of dPCR was demonstrated through the detection of a mutant RAS oncogene. Their use of dPCR differed from ddPCR in the method and degree of sample partitioning [72]. ddPCR has been commercially available since 2011 [73,74] and has been used to successfully detect MRD in leukemia samples and predict relapse [71,75]. ddPCR increases the sensitivity of detection to one blast cell in 10^6^ cells compared to 10^5^ cells in RQ-PCR. Compared to RQ-PCR and RT-PCR, ddPCR is less standardized. Similar to antigen receptor PCR, this method requires patient-specific reagents and is more time consuming and labor-intensive [32]. ddPCR is a promising alternative to traditional methods of PCR because it is more sensitive and can be reliably used for low-target quantitation [76].

### 3.3. Next-Generation Sequencing

Next-generation sequencing (NGS) is a high-throughput sequencing methodology and is a process by which small fragments of DNA are sequenced in parallel multiple times. In a single experiment, NGS can provide accurate data on a DNA sequence and variation information, such as insertions, deletions, rearrangements, and large genomic deletions of exons or whole genes [77]. Sequencing coverage is the average number of reads that cover known reference bases, where the higher the coverage, the higher the accuracy in the sequences that are read [78]. In detecting mutations, approximately 10×–30× depth of coverage is used. NGS has been successfully used to detect MRD in patients with ALL, and [79] it is commonly used to identify epigenetic changes between remission and relapse cells as well as clonal changes in cellular subpopulations in both patients with AML [80] and ALL [81]. Today, commercial sequencing is dominated by several automated commercial methods at a relatively low cost and short processing time.

NGS has often been shown to be more accurate in MRD detection than current universal methods. NGS also provides more in-depth and valuable data on other variations that can be found using genetic sequencing that can be difficult to quantify, and that may lead to a greater likelihood of relapse. Ultimately, this is pertinent in determining a patient’s treatment plan and intensity of therapy. An ongoing clinical trial is using NGS to risk stratify relapsed patients will ALL to a high-risk group who will receive the total body irradiation based preparative regimen prior to allogeneic stem cell transplantation which is associated with long term side effects, and low-risk patients who will receive standard chemotherapy sparing long term toxicities and at the same time maintaining good survival rate [82]. NGS can decrease the occurrence of undetected MRD, which would allow for early intervention, a crucial step to increase ALL patient survival. NGS also has the potential to direct drug development and research, as this technology can be used to identify more genetic patterns associated with relapse, drug resistance, and MRD.

NGS uses PCR for MRD sequencing analysis; however, there are distinctions between RQ-PCR based sequencing and NGS sequencing. RQ-PCR sequencing relies on patient-specific gene analysis through a library of specific primers and personalized primer design for a patient and subsequent sequencing through a second platform, thereby relying on a combination of procedures. In contrast, NGS is performed through a comprehensive analysis through multiplex PCR methods using a large designated primer library with amplicon product sequencing dependent on the sequencing technology of the commercial device used in the combined analysis procedure [83].

NGS involves sonication of the chromosome along with the application of restriction enzymes and then PCR amplification of the fragments on a large platform and comparing the amplicons to a library to match the target sequence. The amplification process can be described as similar to a plasmid vector cloning procedure where the large assortment of DNA fragments generated from the nuclear sonication procedure comprises a large and varied template population that is amplified through PCR and sequenced within the same procedure. Automated NGS applications use primer-based multiplex PCR amplification methods in which a large series of reactions within a procedure amplifies sample gene fragment templates in a microreaction milieu forming clusters of DNA fragments. Colony fragments are then sequenced by cameras that use reporter molecules, such as luciferase, which indicate sequencing progress by light emission and sensor light capture and rapidly record a sequencing reaction one nucleotide at a time. Fragments can also be sequenced by the detection of hydrogen ions released during the polymerization process, among other sequencing processes [84]. Within an experimental procedure, high amounts of coverage of a target sequence are desired, which indicate many independent sequencing reads in a given time frame. A high amount of target reads by light or protons correlates to a quantification of the target strand and allows for computer identification of the target by database comparison. Ultimately, sample cell number quantifications are made based on DNA percentage calculations from sensor computations of the read data during the polymerization process [81,85].

NGS identifies MRD cellular gene fragments, including Ig and TCR variations, fusion genes, insertions, deletions, and other condition-related rearrangements. MRD quantification using NGS can detect MRD cell presence at levels below 10^−5^ (≤0.001%) to a limit of 10^−7^, but low quantification target limits require large bone marrow samples of up to 65 μg. NGS can be performed in a few hours with relative ease compared to PCR and FC due to the high levels of automation.

The advantages of MRD analysis using NGS are that very sensitive detection levels can be obtained using universal primer sets, allowing for the identification of unique targets within one procedure [86]. The disadvantages of NGS include large bioinformatic analysis challenges with low amounts of current laboratory standardization and quality assurance. Procedural difficulties using NGS are partly due to DNA sequence amplification in nonviable cells from a given patient sample [37]. Despite these disadvantages, NGS is able to quantify samples from peripheral blood due to its sensitivity parameters, even though peripheral blood MRD levels are 10-fold less than in the bone marrow [66,67,87,88].

## 4. Discussion

Improved detection methods replacing simple morphology by light microscopy using flow cytometry and PCR were introduced in the 1990s and have allowed for efficient submicroscopic detection of MRD leukemic cells. As these MRD detection methods have become standardized, great improvements in risk stratification and chemotherapy/radiotherapy relapse prognostics have followed, resulting in even more improvement in childhood and adult ALL cure rates.

Partly due to improvements in lymphocyte cellular profiling by FCM and PCR, more intensive treatment (including allogeneic hematopoietic stem cell transplantation and adaptive T cell therapy) based on MRD measurements have been further developed. MRD diagnostic measurements help predict future disease outcomes and act as decision variables in relapse risk and the choice of treatment protocols. Current diagnostic tools are now complemented by improved sequencing technologies and NGS and are increasingly available in clinics providing cellular identification capacities of 10^−7^. PCR and flow cytometry are the standard methods for MRD analyses; however, in the future, it will be important to follow how NGS platforms become more relevant in clinical diagnostics due to the sensitive cell quantification levels these platforms can provide.

## Figures and Tables

**Table 1:** Genetic classification by prognosis of B-cell Acute Lymphoblastic Leukemia.

Good Prognosis	Intermediate Prognosis	Poor Prognosis	Undetermined Prognosis
Hyperdiploid karyotypes	t(1;19); TCF3-PBX1	Hypodiploid karyotypes	t(5;14); IL3-IGH*
t(12;21);ETV6-RUNX1 (TEL-AML1)		t(9;22); BCR-ABL	
		Philadelphia-like ALL	
		11q23 MLL rearrangements	

* t(5;14);IL3-IGH is a World Health Organization classified acute leukemia and prognosis data has not been determined.

**Table 2:** Comparison of MRD detection methods.

	FCM *	Translocation PCR **	Antigen Receptor PCR **	Droplet Digital PCR **	NGS ***
**Turnaround Time**	3–4 h [32]	2–3 days [33]	Weeks [34]	5–8 h [35]	~1 week [36]
**Cost Per Sample**	~$350 [32]	~$500 [33]	~$500 [32]	~500 [32]	~$1000 [32]
**Standardization**	Standardized in different consortia [37]	Limited standardization [37]	Limited standardization [37]	Limited Standardization [32]	Limited Standardization [37]
**Use of Patient-Specific Reagent**	No [37]	No [37]	Yes [37]	Yes [32]	No [37]

* multiparametric flow cytometry; ** polymerase chain reaction; *** next-generation sequencing; BM = bone marrow; PB = peripheral blood.

## Abbreviations

ALL	acute lymphoblastic leukemia
B-ALL	B-cell acute lymphoblastic leukemia
dPCR	digital PCR
ddPCR	droplet digital PCR
FCM	flow cytometry
MRD	minimal residual disease
NGS	next-generation sequencing
PCR	polymerase chain reaction
RQ-PCR	real-time quantitative polymerase chain reaction
RT-PCR	reverse transcription polymerase chain reaction
T-ALL	T-cell acute lymphoblastic leukemia
